# Volume Change in Frontal Cholinergic Structures After Traumatic Brain Injury and Cognitive Outcome

**DOI:** 10.3389/fneur.2020.00832

**Published:** 2020-08-13

**Authors:** Anna Östberg, Christian Ledig, Ari Katila, Henna-Riikka Maanpää, Jussi P. Posti, Riikka Takala, Jussi Tallus, Ben Glocker, Daniel Rueckert, Olli Tenovuo

**Affiliations:** ^1^Division of Clinical Neurosciences, Turku Brain Injury Centre, Turku University Hospital, Turku, Finland; ^2^Department of Neurology, Institute of Clinical Medicine, University of Turku, Turku, Finland; ^3^Department of Neurosurgery, Neurocenter, Turku University Hospital, Turku, Finland; ^4^Department of Computing, Imperial College London, London, United Kingdom; ^5^Department of Perioperative Services, Intensive Care and Pain Medicine, Turku University Hospital, Turku, Finland

**Keywords:** cholinergic system, traumatic brain injury, atlas based volumetric MRI analysis, structural MRI, cognitive testing

## Abstract

The cholinergic nuclei in the basal forebrain innervate frontal cortical structures regulating attention. Our aim was to investigate if cognitive test results measuring attention relate to the longitudinal volume change of cholinergically innervated structures following traumatic brain injury (TBI). During the prospective, observational TBIcare project patients with all severities of TBI (*n* = 114) and controls with acute orthopedic injuries (*n* = 17) were recruited. Head MRI was obtained in both acute (mean 2 weeks post-injury) and late (mean 8 months) time points. T1-weighted 3D MR images were analyzed with an automatic segmentation method to evaluate longitudinal, structural brain volume change. The cognitive outcome was assessed with the Cambridge Neuropsychological Test Automated Battery (CANTAB). Analyses included 16 frontal cortical structures, of which four showed a significant correlation between post-traumatic volume change and the CANTAB test results. The strongest correlation was found between the volume loss of the supplementary motor cortex and motor screening task results (R-sq 0.16, *p* < 0.0001), where poorer test results correlated with greater atrophy. Of the measured sum structures, greater cortical gray matter atrophy rate showed a significant correlation with the poorer CANTAB test results. TBI caused volume loss of frontal cortical structures that are heavily innervated by cholinergic neurons is associated with neuropsychological test results measuring attention.

## Introduction

Traumatic brain injury (TBI) is a significant cause of death and long-term physical and cognitive impairment (1). Despite the broad spectrum of research in TBI during the last decades, both the neuropathology and neurochemistry of post-traumatic deficits are poorly understood (2, 3). The complex nature of TBIs usually involves a combo of focal and diffuse injury pathology and structures (4). Contusions causing focal damage are usually situated in ventral and polar frontal and anterior temporal regions, where the bony ridges of the skull base bruise the brain (5, 6). These regions are important for emotional self-regulation, and social intelligence, which are often defective in patients with TBI. Diffuse axonal injury (DAI) interferes with the inter-neural connections, resulting in slowing and deficits in memory and attention, which are frequently observed in patients with chronic sequelae of TBI (7). It has been suggested that DAI accounts for a greater part of post-traumatic disability compared to focal contusions, and is thus associated with poorer outcome (7, 8).

Cerebral atrophy is a common long-term consequence of TBI (9, 10). The regional pattern of volume loss is not well-characterized, but there are indications that besides to diffuse atrophy there is regionally selective volume loss (9, 11, 12). Voxel based morphometry (VBM) studies have reported a reduction of gray matter volume after TBI in several brain regions, including the frontal and temporal cortices, basal forebrain, hippocampal formation, thalamus, and cerebellum (6, 13, 14). As significant volume loss is often observed in patients with TBI without focal lesions, it seems that atrophy may result from diffuse axonal injury with slowed cell death, more than from straight damage to neuronal cells (15). Studies have reported progressive volume loss from 3 weeks after mild to severe injury up to 12 months, and have also shown that the atrophy proceeds at a greater amount that is seen in natural senescence as far as 3 years post-TBI (16, 17). Numerous studies have demonstrated associations between post-traumatic atrophy, injury severity, and functional outcome (9, 18–20). The relationship with DAI lesion load and post-traumatic atrophy has also been reported (15). Indeed, cerebral atrophy is common as a neuropathological distinctive mark of diffuse injury in the late stage of TBI.

We have shown in our earlier studies using positron emission tomography that the frontal cholinergic system seems to be affected in patients with TBI, and that the degree of cholinergic dysfunction correlates with the therapeutic response for cholinergic drugs (21). Impairments in attention and memory have been well-characterized in TBI. Attention and memory are strongly modulated by cholinergic mechanisms (22). Consequently, there are several lines of evidence that the acetylcholine system is repeatedly related in cognitive impairment after TBI (14, 23, 24). Brain cholinergic neurons and their ascending projections are located anatomically in regions, that are vulnerable in TBI because of the brain anatomy and injury biomechanics (6, 25). VBM studies have shown selective volume loss in structures heavily innervated by cholinergic projections (14). Many reports of pharmacological interventions after TBI are also linear with the malfunction of the acetylcholine pathways (26, 27). Various studies including small patient cohorts have proposed that central acetylcholinesterase (AChE) inhibitors can be affective to cognitive impairment after TBI while being safe (28–33).

Quantitative neuroimaging techniques, initially using computed tomography (CT) and then later using various magnetic resonance imaging (MRI) protocols have provided evidence of volumetric changes in TBI (6, 34). Previous studies have analyzed MRI imaging data based on deformation-based morphometry, VBM, and tensor-based morphometry (11, 33, 34). However, these methods have limitations including difficulties to robustly recognize cortical atrophy with heterogeneous pathology of TBI. Recently developed methodology for automatic MRI segmentation allows improved quantification of brain tissue (9). In this study, we used a novel automated whole-brain segmentation method, which has been described in detail in a previously published article and has been applied successfully in TBI, but also Alzheimer's Disease (AD), and mild cognitive impairment (MCI) studies (35, 36). This method is based on the dependable segmentation of MRI images into many anatomical regions, rather than tissue classes. This method is one of the first consistent segmentation approaches, which is robust to pathology and it segments brain scans into an extensive amount of anatomical structures (20). Automated brain morphometry provides informative measures based on either single time point magnetic resonance (MR) images (structural volumes) or longitudinal MR images (structural atrophy).

The objective of this study was to focus on the anatomical regions of the cholinergic system with automated structural morphometry using T1-weighted MRI in patients with mild to severe TBI. We aimed to assess if there is an association between longitudinal volume change (atrophy rate) of cholinergic regions and cognitive tests of attention.

## Materials and Methods

### Subjects

This study was conducted at the Turku University Hospital, Finland as a part of the EU-funded TBIcare (Evidence-based Diagnostic and Treatment Planning Solution for Traumatic Brain Injuries) project. The TBIcare project was a prospective clinical observational study. All patients were treated according to existing guidelines based on national and international recommendations. New treatment interventions were not evaluated during the data acquisition for this study. Data collection took place between November 2011 to October 2013. Inclusion criteria were as follows: age ≥ 18 years, clinical diagnosis of TBI, and indications for acute head CT according to the National Institute for Health and Care Excellence criteria (http://www.nice.org.uk/guidance/cg176). Exclusion criteria were age <18 years at study entry, blast-induced or penetrating mechanism of TBI, unable to live independently because of a brain disease or other medical condition before the injury, TBI or suspected TBI not needing head CT imaging or not speaking native language. The severity of TBI was classified based on either the lowest non-intubation GCS or the duration of PTA, depending on which one gave higher severity (37): very mild = GCS 15 and PTA <1 h; mild GCS 13–15 and PTA <24 h; moderate GCS 9–12 or PTA >24 h but <1 week; severe GCS 3–8 or PTA >1 week; and very severe PTA >4 weeks. PTA was assessed using the Rivermead method (38). During the project, a total of 141 patients with mild to very severe TBI underwent MRI in the acute-phase (baseline) and/or at the follow-up. In total 120 patients had MRI scans at both time points, but 6 patients were excluded because of image quality problems, and thus the final study group consisted of 114 subjects. Patients with acute orthopedic trauma without any signs of acute head injury, current or earlier brain disorders, or earlier non-trivial head injury were recruited prospectively as a control group (*n* = 40). Of these, 17 patients underwent brain MRI twice at early and late time points, and these were thus included in the analyses of this study.

### Study Design

Study participants were scanned at two time points: during the acute phase of the TBI [mean 20 ± (SD) 15 days] and at the follow-up visit [mean 238 ± (SD) 54 days]. During the follow-up visit, cognitive testing and evaluation of outcome using the Glasgow Outcome Scale Extended (GOSE) (39) were conducted. The control patients were imaged and assessed with equal protocol at the same time points. Structural atrophy occurring between the acute and late time points was quantified with the automated segmentation of MRI method (35). We investigated the volume change in individual regions-of-interest (ROIs) in regions known to have rich cholinergic innervation as well as in larger anatomical regions. Correlation of structural atrophy rates with cognitive tests measuring attention was investigated.

### Image Acquisition

All the study subjects were scanned with Siemens Verio 3T system. For the T1w MR images, a Magnetization-Prepared Rapid Gradient-Echo (MPRAGE) sequence was acquired with parameters: TR 2,300 ms, TE 2.98 ms, TI 900 ms, flip angle 9°, matrix size 256→ 249→ 176 and an isotropic voxel size of 1.0→ 1.0→ 1.0 mm, sagittal slices, using Prescan Normalizer 2D distortion correction and a standard 12 channel head coil.

### Image Processing

Brain segmentation analyses based on T1-weighted MR images were conducted at Imperial College, London, UK. The method of volumetric analyses is described in detail in the earlier work (20, 35, 40). First the images were preprocessed correcting for intensity inhomogeneity with the N4 bias correction algorithm (41). Images were then brain extracted using Pincram (42), which is an iterative, atlas-based method. Each image was then segmented individually using Multi-Atlas Label Propagation with Expectation-Maximization based refinement (MALPEM) (35). As brain atlases, the 30 manually annotated neuromorphometrics anatomical brain atlases were employed. Neuromorphometrics Inc. provided the atlases under academic subscription (http://neuromorphometrics.com/). In MALPEM, the 30 manually annotated anatomical brain atlases are propagated to the image that is to be segmented based on transformations calculated with the robust registration approach MAPER (42, 43). The propagated atlases are then fused into a consensus probabilistic prior estimate using a locally weighted fusion approach, based on the Gaussian-weighted sum of squared distances (GSSD) (35). The GSSD is calculated on images that were intensity normalized using a linear rescaling approach (44). The time-point specific probabilistic segmentation output and the intensity normalized, brain extracted images are then employed to perform the consistent longitudinal segmentation (MALP-EM4D) (35, 45) MALP-EM4D is an approach that employs spatially and temporally varying coupling weights between time-points to obtain temporally consistent segmentation estimates. In the context of TBI, broad structural changes can be expected between both imaging time points. Therefore, the weighted differential bias field correction procedure was used. All brain masks and segmentation results were visually reviewed to ensure reasonable accuracy, taking into consideration the pathology.

All the study subjects were analyzed cross-sectionally at the acute stage of the TBI and longitudinally also analyzing the follow-up image acquired in a later state of TBI, on average 232 days from the injury. Each brain MRI was segmented into 134 distinct atlas labels. When right and left cortical and non-cortical corresponding regions were merged, the dataset included 63 anatomical structures, which had symmetric counterparts in the opposite hemisphere, in total 126 labels (Supplementary Table 1). The remaining eight unpaired structures were: 3rd and 4th ventricle, brain stem, cerebrospinal fluid, optic chiasm, cerebellar vermal lobules I–V, cerebellar vermal lobules VI–VII, and cerebellar vermal lobules VIII–X. From the available regions of this method, we included 16 cortical and 4 non-cortical regions in the analyses. Only structures that are known to associate closely with the cholinergic system were included (46). The basal forebrain cholinergic innervation of the frontal cortex is crucial for attentional performances and as a result, our analyses included the cholinergic nuclei of basal forebrain and brainstem, important cholinergic afferent non-cortical projections (thalamus, ventral diencephalon), and frontal cortical projections (6). We did not include other cortical cholinergic projection areas as our main focus was to investigate atrophy rate correlation with the attentional capabilities that is known to be mainly monitored in frontal regions. We also included in the analyses three large sum structures, to estimate the associations between diffuse atrophy and Cambridge Neuropsychological Test Automated Battery (CANTAB) results. The included sum structures were cortical gray matter (CGM), cerebral white matter, and lateral ventricles. All the included anatomical structures are presented in Table 1.

**Table 1:** Structures selected to analyses.

**Cortical structures**
1) Anterior cingulate gyrus
2) Anterior orbital gyrus
3) Frontal operculum
4) Frontal pole
5) Gyrus rectus
6) Lateral orbital gyrus
7) Medial frontal cortex
8) Middle frontal gyrus
9) Medial orbital gyrus
10) Precentral gyrus medial segment
11) Superior frontal gyrus medial segment
12) Opercular part of the inferior frontal gyrus
13) Orbital part of the inferior frontal gyrus
14) Posterior orbital gyrus
15) Superior frontal gyrus
16) Supplementary motor cortex
**Non-cortical structures**
1) Thalamus
2) Ventral diencephalon
3) Basal forebrain
4) Brainstem
**Sum structures**
1) Cortical gray matter
2) Cerebral white matter
3) Lateral ventricles

For the longitudinal analysis, the structural volumes of all 114 subjects and 17 controls were extracted based on the respective MALP-EM4D segmentations. Atrophy rates were calculated using the logarithmic transform as Δv(t1, t2) = 100%ln(vt2/vt1). Note that the atrophy rate and volume change are used interchangeably, which means that a positive atrophy rate indicates an increase in volume.

### Cognitive Testing and Outcome Assessment

All patients were tested with the CANTAB at the outcome evaluation visit. The functional outcome was assessed using the Glasgow Outcome Scale extended (GOSE) (47, 48). The GOSE provides a widely used measure of recovery after TBI and is given on an ordinal scale from 1 to 8 with a higher score reflecting better outcomes.

From the CANTAB test battery, we included in this study tests known to measure attention, as it is known that sustained attention is mainly monitored by the cholinergic system (49). We used the CANTAB technology providers recommend test battery for attention: (1) rapid visual information processing (RVP), (2) simple reaction time (SRT), and (3) motor screening task (MOT). All patients with TBI finished the MOT, but two patients interrupted SRT (study group *n* = 112), and RVP was failed or interrupted in 11 patients (study group *n* = 103).

### Statistical Analyses

To test for the differences in demographic variables, Chi-Square tests were applied for categorical variables and non-parametric Mann-Whitney *U*-test for continuous variables. Non-parametric Mann-Whitney *U*-test was used to compare regional brain volumes between the groups. Spearman's rank correlation coefficient was used to evaluate if the atrophy rate was associated with CANTAB results. The relationship between CANTAB results and structural atrophy of selected regions was assessed with linear regression models. All the statistical analyses were conducted with the SAS EG (V7.1, SAS Inc., Cary, NC, USA). *P* < 0.05 were considered significant.

### Standard Protocol Approvals, Registrations, and Patient Consents

The Ethical Committee of the Hospital District of Southwest Finland accepted the study protocol. Written informed consent was obtained from all subjects, or where the subject remained unable to give the consent, from the proxy.

## Results

Demographic, imaging, and injury data is summarized in Table 2. This included age, gender, the lowest non-intubated Glasgow Coma Scale (GCS), GOSE, and TBI severity. The control group was comparable with the patients with TBI by age and gender.

**Table 2:** Study population characteristics.

**Subjects**	**Patients (*n* = 114)**	**Controls (*n* = 17)**	
Gender (m/f)	72/42	10/7	*p* = 0.79^a^
Age (median[min; max])	49 (18; 86)	43 (25; 83)	*p* = 0.70^b^
1. scan, day since injury (median[min; max])	20 (1; 51)	n/a	
2. scan, day since injury (median[min; max])	238 (151; 429)	n/a	
GCS (median[min; max])	15 (3; 15)	n/a	
TBI severity (median[min; max])	2 (1; 5)	n/a	
1 = very mild	2		
2 = mild	79		
3 = moderate	18		
4 = severe	12		
5 = very severe	3		
GOSE (median[min; max])	7 (3; 8)	n/a	

a *Chi-square*,

b *Mann-Whitney U-test*.

The age of the patients ranged from 18 to 86 years, average of 48 years. Most patients with TBI had a mild injury (79/114, 69%), and most showed complete or almost complete recovery (GOSE 8–7, 70/114, 61%). Severe injuries were in minority, with severe or very severe injuries in 13% of the patients (15/114). Older patients had a worse outcome by GOSE (*p* < 0.0001).

### Longitudinal Analyses

The analyses included 16 frontal cortical structures, four non-cortical structures, and three sum structures (Table 1). For distinguishing significant longitudinal volume changes, the atrophy rates of patients with TBI were compared to control patients' respective rates. In nine structures the atrophy rates did not differ significantly between the groups and these regions were excluded from further analyses. The excluded structures were various frontal cortical structures and basal forebrain. The results of this analysis are presented in Table 3.

**Table 3:** Atrophy rate differences between TBI subjects and controls.

	**Patients (*n* = 114)**	**Controls (*n* = 17)**	
**Structures**	**mean(SD±) %**		***p***-value^*^
**Cortical structures**			
Anterior cingulate gyrus	−0.78 (6.53)	1.50 (1.62)	**0.0463**
Anterior orbital gyrus	−3.13 (9.65)	0.13 (3.40)	0.1543
Frontal operculum	−0.28 (6.25)	1.02 (2.51)	0.3308
Frontal pole	−1.95 (10.53)	−0.47 (4.03)	0.5331
Gyrus rectus	−4.50 (14.6)	1.23 (2.51)	**0.0045**
Lateral orbital gyrus	−1.76 (8.90)	0.77 (4.15)	0.1563
Medial frontal cortex	−2.04 (9.67)	1.65 (2.78)	**0.0413**
Middle frontal gyrus	0.21 (4.83)	0.83 (3.92)	0.3446
Medial orbital gyrus	−3.42 (8.35)	−0.64 (2.10)	0.0869
Precentral gyrus medial segment	−1.46 (5.99)	2.26 (2.95)	**0.0006**
Superior frontal gyrus medial segment	−1.74 (7.02)	1.02 (3.58)	**0.0289**
Opercular part of the inferior frontal gyrus	−1.41 (5.51)	2.19 (2.83)	**0.0023**
Orbital part of the inferior frontal gyrus	−1.56 (6.289)	1.78 (3.44)	**0.0065**
Posterior orbital gyrus	−1.31 (6.91)	0.30(2.46)	0.1152
Superior frontal gyrus	−0.86 (6.90)	0.82 (5.39)	0.0933
Supplementary motor cortex	−0.99 (5.11)	1.88 (3.01)	**0.0137**
**Non-cortical structures**			
Thalamus	−2.17 (4.23)	0.33 (1.56)	**0.0049**
Ventral diencephalon	−1.29 (3.11)	0.26 (0.94)	**0.0106**
Basal forebrain	2.24 (13.1)	4.60 (7.09)	0.4891
Brainstem	−1.40 (2.79)	0.06 (1.59)	**0.0470**
**Sum structures**			
Cortical gray matter	−0.03 (3.17)	0.97 (1.49)	**0.0156**
Cerebral white matter	−1.21 (2.37)	0.49 (1.49)	**0.0015**
Lateral ventricles	5.99 (14.3)	−2.02 (4.71)	**0.0052**

* *Mann-Whitney U-test. Bold values indicates statistically significant*.

### Cognitive Correlates

The association between longitudinal volume changes and CANTAB test results are presented in Table 4. Generally, the observed associations were rather weak. The strongest correlation was seen between SRT and the supplementary motor cortex atrophy rate (Spearman correlation −0.36, *p* < 0.0001). Also, the volume changes in all non-cortical structures and sum structures had a weak but significant association with the CANTAB results.

**Table 4:** CANTAB results correlation to atrophy rate by Spearman correlation coefficient.

**CANTAB tests**	**MOT**		**SRT**		**RVP**	
	R^*^	***p*-value**	R^*^	***p*-value**	R^*^	***p*-value**
**Cortical structures**						
Anterior cingulate gyrus	−0.11278	0.2322	−0.15377	0.1055	0.08129	0.4143
Gyrus rectus	−0.17595	0.0611	−0.07048	0.4602	0.01688	0.8656
Medial frontal cortex	−0.19303	**0.0396**	−0.18382	**0.0524**	0.16447	0.0969
Precentral gyrus medial segment	−0.20605	**0.0278**	−0.13852	0.1452	0.08524	0.3919
Superior frontal gyrus medial segment	−0.34408	**0.0002**	−0.31293	**0.0008**	0.24127	**0.0141**
Opercular part of the inferior frontal gyrus	−0.29979	**0.0012**	−0.26439	**0.0048**	0.10803	0.2774
Orbital part of the inferior frontal gyrus	−0.01661	0.8608	−0.00912	0.9239	−0.04401	0.6589
Supplementary motor cortex	−0.34610	**0.0002**	−0.36511	**<0.0001**	0.23507	**0.0168**
**Non-cortical structures**						
Thalamus	−0.18057	**0.0545**	−0.18523	**0.0505**	0.21991	**0.0256**
Ventral diencephalon	−0.11895	0.2075	−0.17798	0.0605	0.28386	**0.0037**
Brainstem	−0.16746	0.0749	−0.10309	0.2794	0.23495	**0.0169**
**Sum structures**						
Cortical gray matter	−0.24087	**0.0098**	−0.23797	**0.0115**	0.14312	0.1492
Cerebral white matter	−0.17866	0.0572	−0.25743	**0.0061**	0.22983	**0.0195**
Lateral ventricles	0.20075	**0.0322**	0.21322	**0.0240**	−0.12333	0.2145

*MOT, motor screening task; SRT, simple reaction time; RVP, rapid visual information processing*.

* *Spearman correlation. Bold values indicates statistically significant*.

The structures showing an association between volume loss and CANTAB results were included in linear regression analyses. These consisted of five frontal cortical structures, brain stem, ventral diencephalon, thalamus, cortical gray matter, cerebral white matter, and lateral ventricles. Table 5 presents the results of those brain structures, which showed a significant linear correlation with any CANTAB test. The structures without any significant correlations were excluded from Table 5, but the results of all analyses including non-significant are shown in Supplementary Table 2. The analyses were done for two different groups: first with all patients and then with excluding those with full recovery (GOSE 8). Exclusion of those with GOSE 8 diminished the study group from 114 to 80, but also reduced the number of subjects without longitudinal volume change, and therefore clarified possible existing correlations.

**Table 5:** Relationship between atrophy rate and CANTAB results by linear regression.

	**All GOSE Classes**						**GOSE 3–7**					
	**MOT**		**SRT**		**RVP**		**MOT**		**SRT**		**RVP**	
	***R*-sq**	***p*-value**	***R*-sq**	***p*-value**	***R*-sq**	***p*-value**	***R*-sq**	***p*-value**	***R*-sq**	***p*-value**	***R*-sq**	***p*-value**
**Cortical structures**												
Precentral gyrus medial segment	0.10	**0.0007**	0.03	0.0537	0.00	0.4750	0.15	**0.0003**	0.03	0.1080	0.69	0.4092
Superior frontal gyrus medial segment	0.07	**0.0035**	0.02	0.1322	0.02	0.1182	0.11	**0.0024**	0.02	0.1968	0.04	0.0983
Opercular part of the inferior frontal gyrus	0.08	**0.0019**	0.03	0.0672	0.00	0.4825	0.16	**0.0002**	0.06	**0.0321**	0.00	0.2874
Supplementary motor cortex	0.16	**<0.0001**	0.06	**0.0078**	0.06	**0.0121**	0.26	**<0.0001**	0.07	**0.0212**	0.09	**0.0107**
**Non-cortical structures**												
Thalamus	0.03	0.0805	0.01	0.2939	0.00	0.3730	0.05	**0.0496**	0.01	0.400	0.00	0.64
**Sum structures**												
Cortical gray matter	0.08	**0.0019**	0.02	0.1548	0.01	0.3210	0.15	**0.0005**	0.01	0.1974	0.01	0.3276

*Only structures with significant association presented*.

*MOT, motor screening task; SRT, simple reaction time; RVP, rapid visual information processing. Bold values indicates statistically significant*.

**Figure 1:**
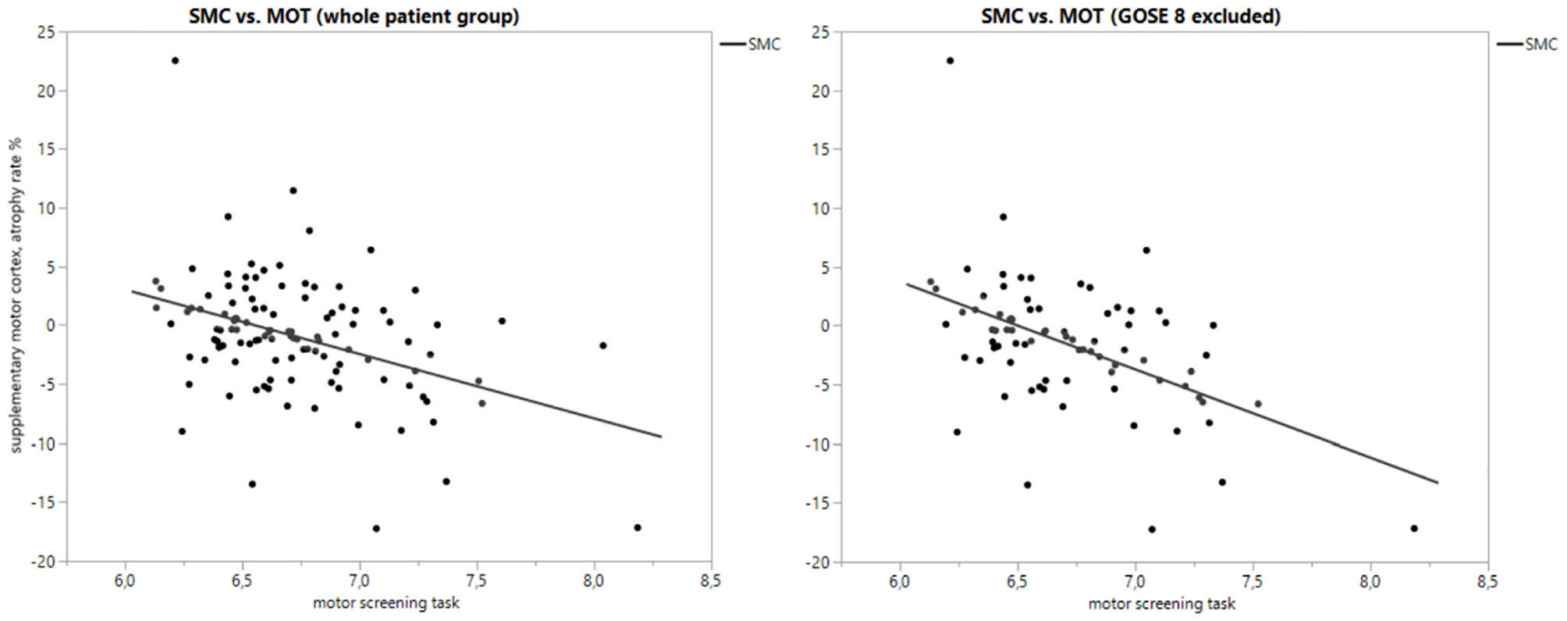
Scatterplot of supplementary motor cortex atrophy rate (%) vs. motor screening task with whole patient group and subgroup.

**Figure 2:**
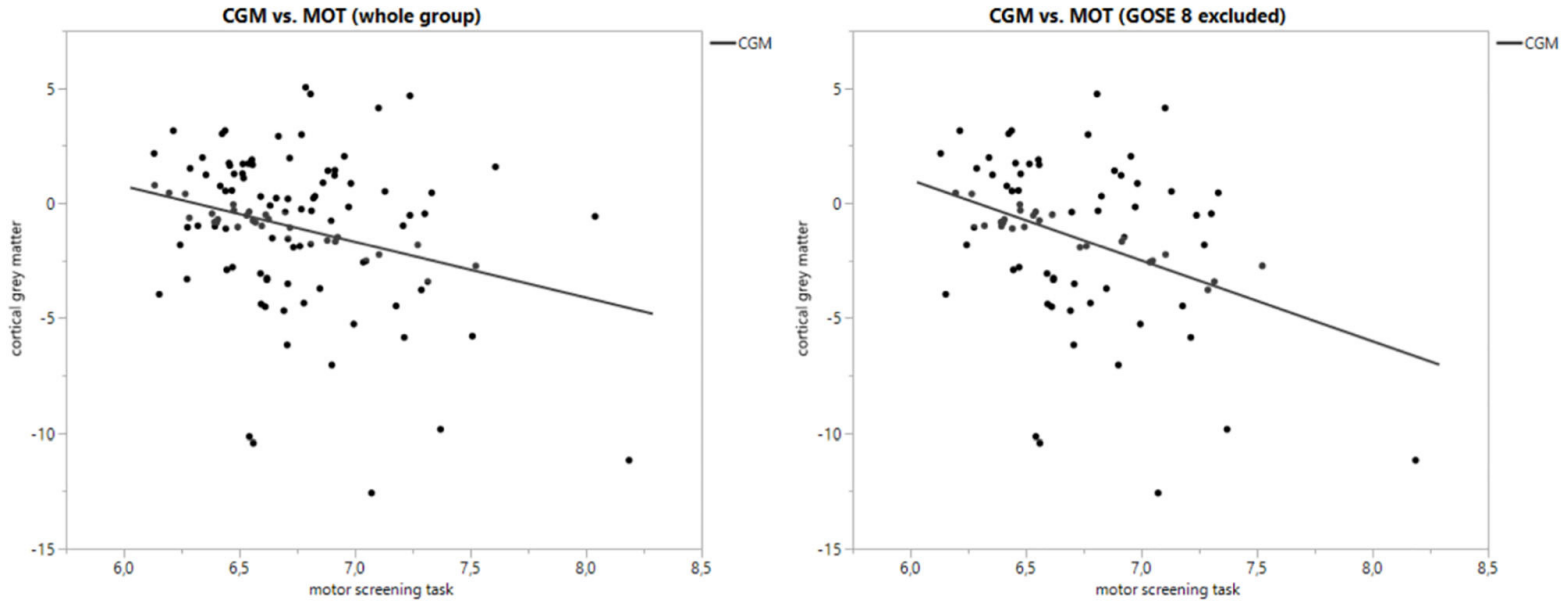
Scatter plot of cortical gray matter atrophy rate (%) vs. motor screening task with whole patient group and subgroup.

The most significant correlation was found between supplementary motor cortex (SMC) atrophy and MOT (all GOSE classes: *F*-ratio = 21.7, *p* ≤ 0.0001; with GOSE 8 excluded: *F*-ratio = 27.0, *p* < 0.0001), where poorer test results were correlated with greater atrophy. Figure 1 shows scatter plots of the linear regression between SMC and MOT, both for the whole group and those with GOSE <8. Also, the volume losses in the premotor cortex, superior frontal gyrus, and inferior frontal gyrus (opercular part) had a significant correlation with MOT results. Of the non-cortical structures, only the thalamus atrophy rate had one significant correlation with a CANTAB test (MOT). The atrophy of thalamus correlated weakly with MOT and significantly only in those with GOSE <8 (*F*-ratio = 3.40, *p* = 0.0496). Of the sum structures measuring diffuse atrophy, the cortical gray matter volume loss correlated significantly with MOT (all GOSE classes: *F*-ratio = 10.2, *p* = 0.0019; GOSE <8: *F*-ratio = 13.2, *p* = 0.0005). Figure 2 shows the linear regression scatter plot between cortical gray matter atrophy rate and MOT.

As in the current study material older patients had a worse outcome, a subgroup analysis was conducted including patients 60 years and older (*n* = 36) to clarify whether the results were driven by the changes in older patients. Results of this subanalysis were in line with the result of the whole group. In linear regression, only MOT from the CANTAB results correlated significantly with the atrophy rates of some regions, detailed results of the analysis are presented in Supplementary Table 3. The only difference to the main analysis was that MOT correlated to the atrophy rate of the brainstem (*F* = 7.00, *p* = 0.0122), and that the correlation between MOT and volume change of thalamus was stronger (*F* = 8.63, *p* = 0.0059). We did also a subgroup analysis including only patients with severe/very severe TBI (*n* = 15), where supplementary motor cortex (*F* = 8.48, *p* = 0.0121) and opercular part of inferior frontal gyrus (*F* = 6.13, *p* = 0.0279) volume loss correlated with MOT. However, these correlations were weaker than in analysis including the whole patient group.

## Discussion

We aimed to investigate if the volume changes after TBI in regions with cholinergic innervation would associate with cognitive test results, especially those measuring attention. Functional MRI studies in humans have shown that the cholinergic system is involved in the attentional activity in frontal structures (6, 50, 51). We found a correlation between CANTAB tests measuring sustained attention and atrophy rates of some brain structures, especially with frontal cortical structures (6). Of all 16 frontal cortical structures, four showed a significant linear correlation with CANTAB test results. Of the non-cortical structures, thalamic volume change associated with motor screening test results, but only in the subgroup of those with incomplete recovery. Of the sum structures measuring volume change of wider brain regions (describing the degree of diffuse atrophy), the cortical gray matter significantly correlated with the MOT results. In the subgroup analysis of subjects 60 years and older, the results were mainly consistent with the whole group.

Mostly the observed associations were fairly weak. In the linear regression analysis, only MOT from CANTAB tests displayed a linear regression to atrophy rates except in supplementary motor cortex, which also correlated significantly with SRT and RVP. One possible reason for this finding may be that SRT and RVP do not solely measure attentional capabilities and consequently brain regions involved in these test performances are likely to be broader than frontal cortical structures and their cholinergic structures. In particular, SRT measures eye-hand coordination whereas RVP demands co-operation with visual cortices in the occipital region, which were not included in our analyses (6).

The fairly weak correlations observed in this study may be due to the patients having mainly mild TBIs when one would expect no remarkable brain volume changes or significant changes in CANTAB test results. We tried to partially avoid this problem by excluding structures without volume loss from further analyses. Also, our linear regression analysis included a subgroup where those with full recovery were excluded from the analyses. On the other hand, the inclusion of only those with moderate to severe TBI or analyzing the outcome classes separately would have led to a notable reduction of patient numbers. Subgroup analysis including only patients with the most severe injuries did not provide any notable additional information, which may be related to the low number of these patients (*n* = 15).

Ascending neuronal projection systems, particularly the cholinergic projections arising from the basal forebrain and brainstem nuclei, have been proposed to contribute to attentional performance by modulating the processing of information in the frontal attentional network (6, 52). Nevertheless, we did not find a correlation between basal forebrain or brainstem atrophy rates and CANTAB results. On the other hand, we were unable to measure directly the cholinergic nuclei volume change, and the regions mentioned used a “sum” structure, which includes all these nuclei. Consequently, the negative result may be partly due to the limitations of the morphometry method used.

Sum structure analyses showed that the cortical gray matter volume change correlated with the MOT results, but the other sum structures (cerebral white matter and lateral ventricles) did not. These sum structures measure primarily diffuse brain atrophy. The reason for the negative result for other structures is unclear, but one might speculate that especially the frontal cortical regions are crucial for attention and therefore the cortical gray matter volume is more strongly associated with an attentional capability.

The used morphometry method may also lead to a bias in volume measurement. As with all automated atlas-based morphometry technics, individual atrophy rates might be distorted as a result of possible segmentation inaccuracies caused by pathological lesions. Atlas-based approaches are limited to the labeling of structures that are represented in the reference atlases and therefor this is an expected phenomenon. On the other hand, the employed method has been proven robust and accurate in the TBI population (19, 34). Also, the results presented here are logical and significant in many structures measured. The explicit segmentations of various pathologies that would allow determining the location of lesions about anatomical structures would be highly necessary for TBI morphometry analysis but are not yet available. This kind of method is under development (53).

The findings of this study are in line with earlier reports. VBM studies in patients with TBI and cognitive sequelae have shown selectively reduced gray matter density in the frontal and temporal cortices, basal forebrain, hippocampal formation, and thalamus (6, 13, 14). Gale et al. found also a strong correlation between gray matter concentration in the frontal and temporal regions and attention (6, 13). Another VBM study showed local atrophy in the amygdala, hippocampus, thalamus, corpus callosum, putamen, precuneus, postcentral gyrus, paracentral lobule, as well as parietal and frontal cortices (9). The structures with the most significant atrophy seem to slightly vary between the studies, probably due to heterogeneity of study populations and TBI severity, but the thalamus and frontal cortical structures appear to be involved in every study like in our current study.

It is unlikely that deficits in cholinergic systems occur independently although this study concentrated selectively on the cholinergic system. Attributing the volume changes to a particular neurotransmitter system is difficult due to the overlapping projections and co-localization of many transmitters. Therefore, we do not claim that the noticed volume alterations would be specific for the cholinergic neurotransmitter system. Nevertheless, either preclinical and human studies have indicated that the cholinergic nuclei, especially in the basal forebrain region, have wide frontal cortical projections and are highly involved in attention maintenance (51, 54). Attentional deficits are also one of the most remarkable and common cognitive sequelae after TBI (55, 56). Our results thus are in line with the theory of cholinergic dysfunction after TBI.

## Data Availability Statement

The raw data supporting the conclusions of this article will be made available by the authors, without undue reservation.

## Ethics Statement

The studies involving human participants were reviewed and approved by the Ethics Committee of the Hospital District of Southwest Finland. The patients/participants provided their written informed consent to participate in this study.

## Author Contributions

AK, H-RM, JP, RT, and JT contributed in patient cohort and study data collection. CL and BG performed the image processing and analysis. OT and DR were involved in planning of the study. OT supervised the work. AÖ performed the statistical analysis, drafted the manuscript, and designed the figures. All authors discussed the results and commented on the manuscript.

## Conflict of Interest

The authors declare that the research was conducted in the absence of any commercial or financial relationships that could be construed as a potential conflict of interest.
