# Supplementary material for: Volume Change in Frontal Cholinergic Structures After Traumatic Brain Injury and Cognitive Outcome
Source: Front Neurol. 2020 Aug 13;11:832. doi: 10.3389/fneur.2020.00832 (PMC7438550; doi:10.3389/fneur.2020.00832)
Supplement: Supplementary file 1 [file Data_Sheet_1.docx]

Supplementary Material

| **Table 1. Cortical and non-cortical structures defined in NMM brain atlas.** | | | |
| --- | --- | --- | --- |
|  |  |  |  |
| Cortical structures | | | Abbreviation |
| 1 | anterior cingulate gyrus | | Acg |
| 2 | anteriior insula | | Ains |
| 3 | anterior orbital gyrus | | AOrG |
| 4 | angulate gyrus | | Ang |
| 5 | calcarine cortex | | Calc |
| 6 | central operculum | | CO |
| 7 | Cuneus |  | Cun |
| 8 | entorhinal area | | Ent |
| 9 | frontal operculum | | FO |
| 10 | frontal pole |  | FRP |
| 11 | fusiform gyrus | | FuG |
| 12 | gyrus rectus |  | Gre |
| 13 | inferior occipital gyrus | | IOG |
| 14 | inferior temporal gyrus | | ITP |
| 15 | lingual gyrus |  | LiG |
| 16 | lateral orbital gyrus | | LOrG |
| 17 | middle cingulate gyrus | | MCgG |
| 18 | medial frontal cortex | | MFC |
| 19 | middle frontal gyrus | | MFG |
| 20 | middle occipital gyrus | | MOG |
| 21 | medial orbital gyrus | | MOrG |
| 22 | postcentral gyrus medial segment | | MPoG |
| 23 | precentral gyrus medial segment | | MPrG |
| 24 | superior frontal gyrus medial segment | | MSFG |
| 25 | middle temporal gyrus | | MTG |
| 26 | occipital pole | | OCP |
| 27 | occipital fusiform gyrus | | OFuG |
| 28 | opercular part of the inferior frontal gyrus | | OpIFG |
| 29 | orbital part of the inferior frontal gyrus | | OrIFG |
| 30 | posterior cingulate gyrus | | PCgG |
| 31 | precuneus |  | Pcu |
| 32 | parahippocampal gyrus | | PHG |
| 33 | posterior insula | | Pins |
| 34 | parietal operculum | | PO |
| 35 | postcentral gyrus | | PoG |
| 36 | posterior orbital gyrus | | POrG |
| 37 | planum polare | | PP |
| 38 | precentral gyrus | | PrG |
| 39 | planum temporale | | PT |
| 40 | subcallosal area | | SCA |
| 41 | superior frontal gyrus | | SFG |
| 42 | supplementary motor cortex | | SMC |
| 43 | supramarginal gyrus | | SMG |
| 44 | superior occipital gyrus | | SOG |
| 45 | superior parietal lobule | | SPL |
| 46 | superior temporal gyrus | | STG |
| 47 | temporal pole | | TMP |
| 48 | triangular part of the inferior frontal gyrus | | TrIFG |
| 49 | transverse temporal gyrus | | TTG |
| **Non-cortical structures** | | |  |
| 50 | 3rd ventricle |  |  |
| 51 | 4th Ventricle |  |  |
| 52 | Accumbens Area | |  |
| 53 | Amygdala |  |  |
| 54 | Brain Stem |  |  |
| 55 | Caudate |  |  |
| 56 | Cerebellum exterior | |  |
| 57 | Cerebellum white matter | |  |
| 58 | Cerebral exterior | |  |
| 59 | Cerebral White Matter | |  |
| 60 | Cerebrospinal fluid | |  |
| 61 | Hippocampus |  |  |
| 62 | Inferior lateral ventricle | |  |
| 63 | Lateral ventricle | |  |
| 64 | Pallidum |  |  |
| 65 | Putamen |  |  |
| 66 | Thalamus |  |  |
| 67 | Ventral diencephalon | |  |
| 68 | Vessel |  |  |
| 69 | Optic chiasm |  |  |
| 70 | Cerebellar vermal lobules I-V | |  |
| 71 | Cerebellar vermal lobules VI-VII | |  |
| 72 | Cerebellar vermal lobules VIII-X | |  |
| 73 | Basal forebrain | |  |

| **Table 2. Relatiolationship between atrophy rate and CANTAB results by linear regression** | | | | | | | | | | | | | | | | | | | |
| --- | --- | --- | --- | --- | --- | --- | --- | --- | --- | --- | --- | --- | --- | --- | --- | --- | --- | --- | --- |
|  |  |  | **ALL GOSE CLASSES** | | | | | | | |  | **GOSE 3-7** | | | | | | | |
|  |  |  | MOT | |  | SRT | |  | RVP | |  | MOT | |  | SRT | |  | RVP | |
|  |  |  | F-ratio | p-value |  | F-ratio | p-value |  | F-ratio | p-value |  | F-ratio | p-value |  | F-ratio | p-value |  | F-ratio | p-value |
| **Cortical structures:** | |  |  |  |  |  |  |  |  |  |  |  |  |  |  |  |  |  |  |
| Medial frontal cortex | | | 1.20 | 0.1688 |  | 0.73 | 0.1180 |  | 0.67 | 0.1781 |  | 1.55 | 0.2173 |  | 0.002 | 0.9888 |  | 0.55 | 0.4486 |
| Precentral gyrus medial segment | | | 12.14 | 0.0007 |  | 3.80 | 0.0537 |  | 0.50 | 0.4750 |  | 14.11 | 0.0003 |  | 2.65 | 0.1080 |  | 0.69 | 0.4092 |
| Superior frontal gyrus medial segment | | | 8.89 | 0.0035 |  | 2.30 | 0.1322 |  | 2.48 | 0.1182 |  | 9.83 | 0.0024 |  | 1.70 | 0.1968 |  | 2.81 | 0.0983 |
| Opercular part of inferior frontal gyrus | | | 10.09 | 0.0019 |  | 3.41 | 0.0672 |  | 0.50 | 0.4825 |  | 14.85 | 0.0002 |  | 4.77 | 0.0321 |  | 0.60 | 0.2874 |
| supplementary motor cortex | |  | 21.67 | <.0001 |  | 7.35 | 0.0078 |  | 6.53 | 0.0121 |  | 27.0 | <.0001 |  | 5.54 | 0.212 |  | 6.89 | 0.0107 |
| **Non-cortical structures:** | |  |  |  |  |  |  |  |  |  |  |  |  |  |  |  |  |  |  |
| Thalamus |  |  | 3.11 | 0.0805 |  | 1.11 | 0.2939 |  | 0.81 | 0.3730 |  | 3.40 | 0.0496 |  | 0.72 | 0.400 |  | 0.64 | 0.1413 |
| Ventral diencephalon | | | 1.19 | 0.1883 |  | 1.16 | 0.2842 |  | 0.58 | 0.4467 |  | 1.59 | 0.2108 |  | 0.57 | 0.4537 |  | 0.14 | 0.7081 |
| Brainstem | | | 2.84 | 0.0948 |  | 0.93 | 0.8871 |  | 1.72 | 0.1923 |  | 3.11 | 0.0815 |  | 0.50 | 0.4565 |  | 0.98 | 0.3244 |
| **Sum structures:** | |  |  |  |  |  |  |  |  |  |  |  |  |  |  |  |  |  |  |
| cortical grey matter | |  | 10.15 | 0.0019 |  | 2.05 | 0.1548 |  | 0.99 | 0.3210 |  | 13.21 | 0.0005 |  | 1.69 | 0.1974 |  | 0.97 | 0.3276 |
| cerabral whitematter | | | 3.65 | 0.0585 |  | 1.99 | 0.1609 |  | 1.20 | 0.2756 |  | 3.74 | 0.0567 |  | 1.47 | 0.2287 |  | 0.78 | 0.3788 |
| lateral ventricles | | | 2.51 | 0.1162 |  | 0.74 | 0.1548 |  | 0.03 | 0.8532 |  | 2.66 | 0.1069 |  | 0.63 | 0.2201 |  | 0.02 | 0.8843 |
| MOT=motor screening task, SRT= simple reaction time, RVP= rapid visual information processing | | | | | | | | | | |  |  |  |  |  |  |  |  |  |

| **Table 3. Relatiolationship between atrophy rate and MOT results by linear regression** | | | | | |
| --- | --- | --- | --- | --- | --- |
| Subgroup analysis with patient 60 ≤ years (n=36) | | | | | |
|  |  |  | **MOT** | | |
|  |  |  | F-ratio | R_sq_ | p-value |
| **Cortical structures:** | |  |  |  |  |
| Medial frontal cortex | | | 2.78 | 0.0755 | 0.1049 |
| Precentral gyrus medial segment | | | 11.72 | 0.2564 | 0.0016 |
| Superior frontal gyrus medial segment | | | 3.31 | 0.0888 | 0.0776 |
| Opercular part of inferior frontal gyrus | | | 6.73 | 0.1652 | 0.0139 |
| Supplementary motor cortex | |  | 14.85 | 0.3039 | 0.0005 |
| **Non-cortical structures:** | |  |  |  |  |
| Thalamus |  |  | 8.63 | 0.2024 | 0.0059 |
| Ventral diencephalon | | | 2.63 | 0.0802 | 0.0943 |
| Brainstem | | | 7.00 | 0.1708 | 0.0122 |
| **Sum structures:** | |  |  |  |  |
| cortical grey matter | |  | 9.23 | 0.2134 | 0.0046 |
| cerabral whitematter | | | 2.65 | 0.0722 | 0.1130 |
| lateral ventricles | | | 2.99 | 0.0810 | 0.0924 |
| MOT=motor screening task | |  |  |  |  |
